# Influence of Various Coal Energy Wastes and Foaming Agents on Foamed Geopolymer Materials’ Synthesis

**DOI:** 10.3390/ma16010264

**Published:** 2022-12-27

**Authors:** Elena A. Yatsenko, Boris M. Goltsman, Sergei V. Trofimov, Yuri V. Novikov, Victoria A. Smoliy, Anna V. Ryabova, Lyudmila V. Klimova

**Affiliations:** Department “General Chemistry and Technology Silicates”, Platov South-Russian State Polytechnic University (NPI), Prosveshcheniya Street 132, Rostov Region, 346428 Novocherkassk, Russia

**Keywords:** foamed geopolymer, alkali-activated materials, coal energy waste, foaming agent, cellular structure formation

## Abstract

The regularities of obtaining foamed alkali-activated geopolymer materials based on different wastes of coal power engineering (fly ash, fuel (boiler) slag, ash, and slag mixture) were considered. The phase composition of the studied waste showed the presence of a significant amount of the amorphous phase, as well as a crystalline phase. mostly in the form of high quartz. The microstructure of studied the waste showed that the fly ash consisted of monodisperse hollow aluminosilicate microspheres, the fuel slag was represented by polydisperse irregular particles, and the ash and slag mixture included both of these materials in different ratios. Blowing agents such as aluminum powder, hydrogen peroxide, and sodium hypochlorite were chosen to achieve the porous structure of the geopolymer materials. The calculations of the geopolymer precursor compositions were carried out. Samples were synthesized, and their physical and mechanical properties, such as density, strength, porosity, and thermal conductivity, were analyzed. The micro- and macrostructure of the samples, as well as the pore distribution of the obtained geopolymers were studied. Conclusions were made on the choice of the most-optimal foaming agent and the optimal coal combustion waste suitable for the synthesis of the geopolymer materials.

## 1. Introduction

Coal-fired energy generation has been the backbone of the global electric power industry for many years. According to the International Energy Agency (IEA), the share of coal energy generation in 2020 was 35.2%. This means that coal provides more than one third of the world’s electricity production. At the same time, global coal energy generation reached record levels in 2021, increasing the amount of emissions, not only of CO_2_, but also of a number of solid mineral wastes, such as fly ash, boiler slag, and ash and slag mixture (ASM). It is known that the total volume of coal energy waste annually reaches 1 billion tons [1].

The world’s processing of waste from coal energy generation is very great. China annually generates over 200 million tons of ash and slag waste, and the volume of their processing reaches 65%. They are mainly used for the needs of the cement industry, in the production of inexpensive building materials, as a concrete additive in construction [2,3]. In India, about 215 million tons of ash and slag waste is generated annually, and the volume of their processing is also 65%. The main consumers of ash and slag in this country are the cement industry, the road sector, the ceramic industry, and agriculture, where they are used as ameliorants, etc. [4].

In the Russian Federation, the problem of coal energy waste accumulation is the most acute. With the annual formation of more than 22 million tons of ash and slag waste, the volume of their processing is small (no more than 10–12%) [5]. According to various estimates, the volume of accumulated waste at ash–slag dumps is 1.4–1.8 billion tons in an area of more than 20,000 km^2^ [6,7]. Ash–slag dumps are hydraulic structures surrounded by fencing dams, where the ASM is supplied by pulp-pipe systems. At the same time, dumps are designed directly near the territory of the power plant and, accordingly, near residential areas. Taken together, ash–slag dumps and the waste stored in them are objects of increasing danger, and therefore, the search for new ways for their disposal is relevant.

Coal energy is the source of three types of waste: ASM, fly ash, and boiler slag. ASM is a polydisperse material, which contains 45–60% SiO_2_, 10–30% Al_2_O_3_, and the compounds of calcium, magnesium, sulfur, and iron [7,8]. Fly ash is a highly dispersed material consisting of spherical particles represented by hollow aluminosilicate spheres with a diameter of 0.1–100 µm [9,10]. It is known that fly ash can be used as an additive in cement, concrete, mortar, lime–pozzolanic mixtures, etc. [11]. In addition, fly ash is used in road construction, dams, agriculture, and the paint industry [12]. 

Fuel (boiler) slag is an amorphous vitreous material, represented by the non-combustible mineral part of coal. It is formed during a high-temperature combustion (at temperatures above 1300 °C) in the boilers of thermal power plants and the cooling of the resulting melt [13,14]. Unlike fly ash, fuel slags are formed at higher temperatures and do not contain residues of unburned coal. Thus, it is a very homogeneous material, but its chemical composition is highly dependent on the coal composition and can vary over a wide range.

For example, in the study [15], the authors considered the production of geopolymer materials based on metakaolin, ground granules of blast furnace slag and fly ash, activated with an alkaline activator solution consisting of sodium silicate and sodium hydroxide. This group of authors found that the studied wastes used as precursors for the production of geopolymers are a reliable alternative to Portland cement, especially fly ash, due to the high content of aluminosilicates in their composition.

In the study [16], the authors proposed a sustainable roadmap for the additive production of geopolymers and their use in the construction industry. The authors considered the process of 3D printing of objects from geopolymer materials. It has been suggested that geopolymers are more suitable for this purpose than traditional Portland cement.

The presence of a high proportion of the amorphous aluminosilicate phase in these materials makes coal energy waste a promising raw material for obtaining a new class of materials—geopolymers—which are hydraulic binders of alkaline activation [17,18,19]. The term “geopolymer” was first proposed by the French chemist and materials scientist Joseph Davidovits in 1978 [20]. At the same time, in the early stages of the development of geopolymer material technology, the main raw material for their production was metakaolin, which was subjected to high-temperature processing. The material obtained in this way was very expensive and not very competitive in comparison with it counterparts. Further research made it possible to study the prospects for the use of geopolymer materials in the recycling of large-tonnage wastes of coal energy [21,22,23,24,25]. 

Geopolymers are glass–crystalline aluminosilicate materials consisting of [SiO_4_] and [AlO_4_] tetrahedra connected in series in chains and rings, forming two- and three-dimensional structures. Despite the large number of questions associated with the process of geopolymerization, it was discovered that it proceeds in three stages [20]:

1. Dissolution of SiO_2_ and Al_2_O_3_ in an alkaline medium—a concentrated solution of sodium hydroxide or potassium hydroxide;
2. Destruction of polymer structures in the initial raw materials of natural or technogenic origin;
3. Solidification and compaction of the material due to the polymerization of the monomers formed in the second stage.

The empirical formula of the geopolymer is expressed by (1):

M_n_{(SiO_2_)_z_AlO_2_}_n_,wH_2_O (1)

where M—an alkali metal atom, n—the degree of polymerization or polycondensation, and z—the Si/Al ratio equal to 1, 2, or 3, which determines the type of geopolymer material. At the ratio Si/Al = 1, the structure is called polysialate, at the ratio Si/Al = 2, polysialate-siloxo, and at the ratio Si/Al = 3, polysialate-disiloxo [26,27].

Foamed geopolymer materials with a porous (cellular) structure, low thermal conductivity, and a density of less than 1000 kg/m^3^ are very promising. The cellular structure of geopolymer materials is achieved by introducing additives—foaming agents that react with the components of the raw geopolymer mixture or decompose under its influence. This addition of additives leads to the formation of gases, the raw mixture foaming, and subsequent drying and hardening with the formation of a solid foam [28,29,30,31]. Foaming agents must allow obtaining a uniformly distributed porous structure, which provides the high physical and mechanical characteristics of the foamed material. To obtain foamed geopolymer materials, a number of foaming agents can be used, which are individual substances or mixtures thereof, such as aluminum powder, hydrogen peroxide, sodium hypochlorite, mixtures of aluminum nitride and iron (II) sulfite, sodium perborate, silicon carbide, etc. [32].

Different aluminosilicate materials could be used as raw materials for geopolymer synthesis. They could be both of natural (metakaolin, feldspars, clay minerals, volcanic ash, etc.) or artificial (ash and slag mixtures, coal mining waste, metallurgical production waste, glass production waste, construction waste, etc.) origin. The use of waste as a raw material is more promising since it leads to the recycling of waste and the cost reduction of the obtained materials.

Thus, the purpose of this work is to study the possibilities of obtaining foamed geopolymer materials using different wastes from coal combustion at the Novocherkassk State District Power Plant, the largest coal-fired power plant in the South of the Russian Federation.

The scientific novelty of this study is in the analysis of the influence of different coal combustion wastes and different foaming agents on the structure formation and foaming of geopolymer precursors. This analysis is also important because most of the articles deal with one specific type of raw material for the synthesis of geopolymers (mostly fly ash). However, only a few studies use ash and slag mixtures and fuel slag for this purpose. The same situation occurs for the comparison of different foaming agents. Therefore, geopolymer synthesis using various silicate raw materials and foaming agents could reveal its main peculiarities. Consequently, it will allow large-scale recycling of waste into construction materials. Foamed geopolymer materials can be used in the construction of buildings as a heat-insulating layer, in the design of heat-insulating layers of reservoirs and pipelines, in road construction [33,34], as sorbents and filters [35,36], etc.

## 2. Materials and Methods

### 2.1. Materials

To identify the regularities of the synthesis of foamed geopolymer materials, mineral waste from the coal energy production of the Novocherkassk State District Power Plant (Novocherkassk, Rostov Region, Russia, fuel: anthracite stump coal from the Donetsk coal basin) was used as an aluminosilicate precursor, namely: ASM, fly ash, and fuel slag. It is known that the important characteristics that affect the final technical and operational properties of foamed geopolymer materials are the humidity, chemical, and phase composition of the precursor and particle size [37,38,39]. Therefore, the raw materials listed above were dried to a constant weight (3 successive measurements differed by no more than 0.1 wt. %). Further, to increase the reactivity, the ash and slag aluminosilicate component (precursor) was milled to a particle size of less than 250 μm.

A mixture of waterglass (sodium hydrosilicate, silicate modulus = 2, water content 52 wt. %, Sil-Ex, Asbest, Russia) and an alkaline solution of NaOH were used for alkaline activation of the aluminosilicate components. As previous studies have shown [40,41,42], the optimal molar concentration of the NaOH solution is in the range of 8 –12 M, since its further increase reduces the resulting compressive strength of the foamed geopolymer materials. Probably, this effect is achieved due to an increase in the concentration of hydroxide ions, which helps to limit their mobility and slow down the formation of coagulation structures [43]. The preparation of an alkaline solution was performed in a separate vessel, where a pre-weighed sample of NaOH powder with a purity of 99% (LenReactive, St. Petersburg, Russia) was dissolved in distilled water to obtain a molar concentration of 12 mol/L.

Spherical, dispersed aluminum (powder) of the ASD-1 grade with a purity of 99% and a specific surface area of 148 m^2^/g (GC Metal Energo Holding, Ekaterinburg, Russia), 30% hydrogen peroxide solution (LenReactive, St. Petersburg, Russia), and sodium hypochlorite of the A grade (LenReactive, St. Petersburg, Russia) were used as the foaming agents. Aluminum powder is a fine, highly dispersed silver-colored metal powder with a particle size of 10–30 µm, which, as a rule, contains a small amount of impurities such as copper, silicon, iron, and manganese. This type of powder is obtained by spraying molten aluminum. The aqueous solution of hydrogen peroxide is a colorless, odorless, heavy polar liquid. This is an inorganic substance, a compound of hydrogen and oxygen, the simplest representative of the class of peroxides. Due to hydrogen bonds, hydrogen peroxide molecules are strongly associated; therefore, hydrogen peroxide has higher values of density, viscosity, and boiling point (compared to water), which vary depending on its concentration. The sodium hypochlorite solution is a colorless liquid with a characteristic chlorine odor. Sodium hypochlorite is an inorganic sodium salt of hypochlorous acid, which is an unstable white crystalline substance, which is highly soluble in water (under normal conditions: 53.4 g per 100 g of water). Sodium hypochlorite is a large-scale product of the chemical industry obtained by brine electrolysis or by the chlorination of sodium hydroxide solutions.

### 2.2. Methods

The determination of the weight percentage of fractions in ASM and fly ash was performed using an AC-200U impact sieve analyzer (NPK Mekhanobr-tekhnika, St. Petersburg, Russia). A sample of the material was placed in the analyzer for 10 min, after which, each fraction was weighed and its percentage was calculated.

Qualitative X-ray phase analysis of the precursors was carried out on an ARLX’TRA diffractometer (Thermo Fisher Scientific, Waltham, Massachusetts, USA) with beam focusing for reflection according to the Bragg–Brentano method. The characteristic radiation of a copper anode (wavelengths CuKα1 1.5406 Å, CuKα2 1.5444 Å) at a voltage of 35 kV and an anode current of 30 mA was used in the study. The diffraction pattern was recorded at survey angles of 5–60° (2 θ) with a step of 0.04°. The data interpretation was carried out using the Crystallographica Search-Match Version 3 software package of the ICDD PDF 2 database (International Center for Diffraction Data). Semi-quantitative analysis was performed using the Materials Analysis Using Diffraction (MAUD) software. [44], based on the Rietveld method. The obtained X-ray diffraction patterns were optimized using the built-in least-squares algorithm, after which the phases’ concentrations were determined. X-ray phase analysis was carried out at the Collective Use Center “Nanotechnologies” of Platov South-Russian State Polytechnic University (NPI) (Novocherkassk, Rostov region, Russia).

The determination of the main oxides’ concentration was carried out by X-ray spectral fluorescence analysis (XRF) on a sequential vacuum spectrometer (with wavelength dispersion) Model Axios mAX (PANalytical, Almelo, Netherlands). The spectrometer is equipped with a 4 kW X-ray tube with a Rh anode, maximum tube voltage 60 kV, and anode current 160 mA. The analysis was performed at the Collective Use Center of the Institute of Geology of Ore Deposits, Petrography, Mineralogy and Geochemistry of the Russian Academy of Sciences (Moscow, Russia).

The analysis of the microstructure coal energy waste was performed using a scanning electron microscope TESCAN VEGA (Tescan Orsay Holding, Kohoutovice, Czech Republic) with a thermionic tungsten cathode, a 6 μm beam diameter falling on the sample, and a voltage of 20 kV. The analysis of the microstructure of the geopolymers was performed using a JEOL JSL 5300 scanning electron microscope (JEOL, Tokyo, Japan) operating at 20 kV, configured to use secondary electron backscattering detectors. This equipment is a part of the “Nanotechnologies” CCU of the Platov South-Russian State Polytechnic University (NPI). 

The thermal conductivity of the synthesized samples was determined using a thermal conductivity meter (ITP-MG4’100/Zond’, SKB StroyPribor, Chelyabinsk, Chelyabinsk region, Russia) by the method of stationary heat flow passing through a sample of a certain thickness directly perpendicular to the sample faces. The device measures the thickness of the sample, the heat flow density, and the temperature of the opposite front faces and, then, calculates the effective thermal conductivity λ, W/(m·K), according to Equation (2).

λ = (H·q)/(T_H_ − T_C_), W/(m·K) (2)

where λ—the effective thermal conductivity, W/(m·K); q—the density of stationary heat flow passing through the measured sample, W/m^2^; T_H_—the temperature of the hot edge of the measured sample, K; T_C_—the temperature of the cold edge of the measured sample, K.

The linear dimensions of the synthesized samples after curing were determined with an electronic caliper (WDK-MD15001, WiederKraft Rus, St. Petersburg, Russia) with a measurement accuracy of ±0.03 mm, after which the sample volume was calculated by multiplying the length of the geopolymer by its width and height. The mass of the samples was measured with analytical laboratory scales (PR224, OHAUS, OHAUS Co., Parsippany, New Jersey, USA) with a measurement accuracy of 0.0001 g. The density of the samples d, kg/m^3^, was determined as the ratio of the mass to the volume of the sample according to Equation (3):

d = m/V · 1000, kg/m^3^ (3)

where m—sample mass, g; V—sample volume, cm^3^.

Porosity P, %, shows the volume of pores in a porous material, which is defined as the ratio of the bulk density d_b_ to the true density d_t_ of the synthesized geopolymer. Porosity was calculated according to Equation (4):

P = (1 − d_b_/d_t_) · 100, % (4)

where d_b_—sample bulk density, kg/m^3^; d_t_—sample true density, kg/m^3^.

The maximum compressive strength R, MPa, was determined using a test press (TP-1–350, TestPress, Misailovo village, Russia) with a force measurement range of 0.1 to 350 kN with a measurement accuracy of ±2% in the range of 0.1 to 7 kN, and ±1%—from 7 to 350 kN. The maximum compressive strength was calculated according to Equation (5):

R = 1000 · P/S, MPa(5)

where P—breaking load, kN; S—sample area, m^2^.

Each recorded test value was the medium of 3 measurements.

The pore size and distribution were determined automatically using the open-access ImageJ software [45]. To determine the pore size, the Feret diameter was used, which is characterized as the largest distance between two points located within the boundaries of the pores. A reflected-light geological ore microscope (Polar 1, Micromed, Ningbo, China) with a digital video eyepiece (ToupCam UCMOS05100KPA, Hangzhou ToupTek Photonics Co, Hangzhou, China) was used for the microphotography of sample surfaces at high resolution.

### 2.3. Synthesis of Foamed Geopolymer Materials

**Figure 1:**
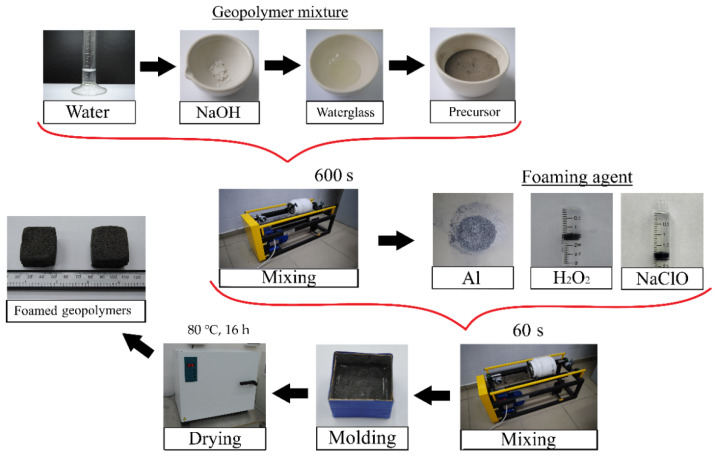
Technology of foamed geopolymer materials’ synthesis.

As mentioned above, fly ash, fuel slag, and ASM were used as the precursors. ASD-1-grade spherical dispersed aluminum (powder), 30% hydrogen peroxide solution, and Grade A 10% sodium hypochlorite solution were used as the foaming agents. Therefore, the following labelling was chosen: Composition No. 1 used fly ash as the precursor, No. 2, fuel slag, and No. 3, ASM. The letter designations indicate the foaming agent in the form of: a—spherical dispersed aluminum, h—30% hydrogen peroxide solution, and s—sodium hypochlorite solution. For example, “2h” is the raw mixture based on the slag precursor and the 30% hydrogen peroxide solution as a foaming agent. The component compositions of the raw mixtures are shown in Table 1, and the technological scheme for the synthesis of the foamed geopolymer materials is shown in Figure 1.

The technology for the synthesis of the foamed geopolymer materials consisted of the following stages: dried aluminosilicate precursors were crushed to a particle size of less than 250 µm. A mixture of waterglass and NaOH solution was used as an activating agent for the alkaline activation of the aluminosilicate precursor. Water was added to a given amount of NaOH powder to obtain a 12 M solution. The resulting solution was mixed with a sample of sodium waterglass, and the resulting suspension was poured into a weighted precursor powder. The geopolymer suspension was stirred for 600 s in a drum mill (MSL-1S, PromStroyMash, Kaluga, Kaluga region, Russia) at 120 rpm in a ceramic drum with the ratio “geopolymer mixture:grinding media” = 1:1.5. After the mixture was prepared, the required foaming agents were added to the compositions (above 100%), and the mixture was stirred for 60 s under the same conditions.

The resulting compositions were poured into cubic molds with an edge length of 30 mm and sent for drying and curing. One-stage drying in an SS-80-01 SPU oven (Smolenskoye SKTB SPU, Smolensk, Smolensk region, Russia) at a temperature of 80 °C and a curing time of 16 h was chosen as the temperature–time mode [46].

## 3. Results and Discussion

### 3.1. Physical and Chemical Studies of Coal Energy Wastes

The chemical composition of the coal energy wastes was determined in order to determine the possibility of using them as the main raw material for the production of the geopolymer materials. Table 2 shows the chemical composition of the coal energy wastes used in the research. Furthermore, the indicators of the raw materials’ quality were calculated (Table 3). The silicate modulus characterizes the content of silicate minerals in the main raw material. It can be presented as the ratio of the SiO_2_ that reacts with other oxides to the total content of Al_2_O_3_ and Fe_2_O_3_. Materials with a high silicate modulus form an unstable amorphous structure upon solidification. Furthermore, with an increased value of the silicate modulus, the material hardens slowly and has greater strength. The basicity modulus is a characteristic of the activity of the raw materials used and their stability during lime decomposition. When Mb < 1, the raw materials are classified as acidic and not prone to lime decomposition. When Mb > 1, the raw materials are classified as basic and prone to lime decomposition. The quality coefficient evaluates hydraulic activity and expresses the ability of binders to continue hardening and retain strength in an aqueous medium (after preliminary hardening in air). The results of the calculations are presented in Table 3.

Table 2 shows that the studied materials mainly consisted of SiO_2_ and Al_2_O_3_; all three materials contained a significant content of Fe_2_O_3_. Fly ash is usually divided into two classes: high-calcium (CaO content of more than 10%) and low-calcium (CaO content of less than 10%). According to the chemical composition, the studied fly ash contained 2.03% CaO, and so, it is low-calcium. It is suitable for geopolymer raw materials because the high content of calcium oxide impairs the course of geopolymerization reactions and iimpairs the microstructure and workability of the raw mix [47].

The calculated quality indicators (Table 3) clarified the suitability of the studied wastes as binders and fillers. The silicate modulus of all studied wastes was at the level of 1.7. This means that the solidification of such mixtures led to the formation of a stable solid structure. The basicity modulus of all three raw materials was less than 1.0, and therefore, these materials are acidic. The quality coefficient of the studied materials shows that they have low hydraulic activity. Thus, the studied wastes do not have binding properties, but they can be used as an aluminosilicate framework in the formation of a geopolymer structure.

**Figure 2:**
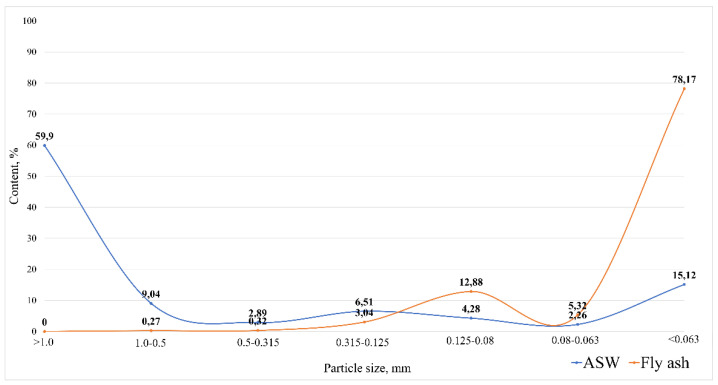
Particle size distribution diagrams.

Sieve analysis was performed to determine the particle size distribution of the precursors and to identify the requirements for their subsequent processing. Since fuel slag is represented by vitreous pieces larger than 5 cm and its dimensions depend on the initial grinding, it was decided not to conduct studies to determine its fractional composition. Particle size distribution diagrams of ASM and fly ash are shown in Figure 2. 

As can be seen from Figure 2, ASM is dominated (60%) by a large fraction with a particle size of more than 1 mm, mainly represented by lumpy fuel slag. Thus, the ash and slag mixture is a polydisperse material that requires further grinding and milling, because the finely dispersed material is more reactive. Fly ash is a highly dispersed material with a predominance of particles smaller than 0.063 mm (78.2%), which allows the use of this material without additional milling.

**Figure 3:**
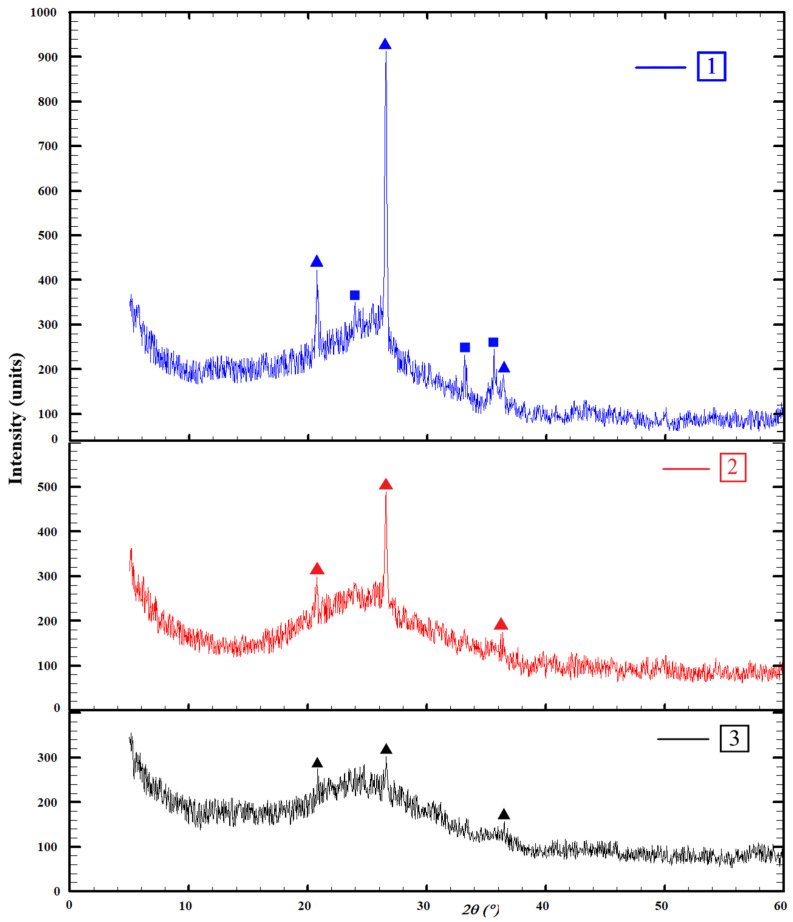
Results of X-ray analysis of coal energy waste: 1—ASM; 2—fly ash; 3—slag; Δ—high quartz (SiO_2_), □—hematite (Fe_2_O_3_).

X-ray phase analysis was carried out in order to identify crystalline peaks and, thus, to identify the phases or minerals present in the material. The obtained X-ray patterns of the studied coal energy wastes are presented in Figure 3. 

Figure shows that all studied X-ray patterns are characterized by the presence of a ferroaluminosilicate amorphous glass phase, which is confirmed by an amorphous “halo” at the shooting angles of 18–38° (2 θ). It forms due to slag quenching in water and a consequent sharp decrease in the temperature. Therefore, the aluminosilicate melt does not have time to crystallize, and the glass phase is formed. All the studied wastes consist of the same crystalline phases in the form of high quartz (PDF card: 46-1045) and hematite (PDF card: 33-0664) (at the limit of device sensitivity). The highest intensity of the crystalline peaks corresponds to the ASM sample. This is probably due to the fact that part of the slag is taken from the boiler, and the rest is mixed with water, after which, it is sent to the ash–slag dump by hydrotransportation. Then, the deposition of ASM on the dump leads to its contamination with different natural materials: sand, clay, etc. This leads to a higher crystalline intensity and worse stability.

The semi-quantitative X-ray phase analysis was performed using the Maud software (Ver.2.992, University of Trento, Trento, Italy). The ash and slag wastes are represented by an amorphous phase of 72.86 ± 0.72% and a crystalline phase of 27.14% (20.39 ± 0.84% of high quartz and 6.75 ± 0.02% of hematite). Fly ash is represented by an amorphous phase of 81.61 ± 0.47% and a crystalline phase of 18.39% (high quartz). Fuel slag is represented by an amorphous phase of 98.89 ± 0.03% and a crystalline phase of 1.12% (high quartz).

It is known that an amorphous structure has excess internal energy, which is why it has excellent reactivity and a high intensity of interaction with an alkaline activator. Therefore, the increased content of the amorphous phase in raw materials, theoretically, should have a positive effect on the final physical–chemical and operational properties.

**Figure 4:**
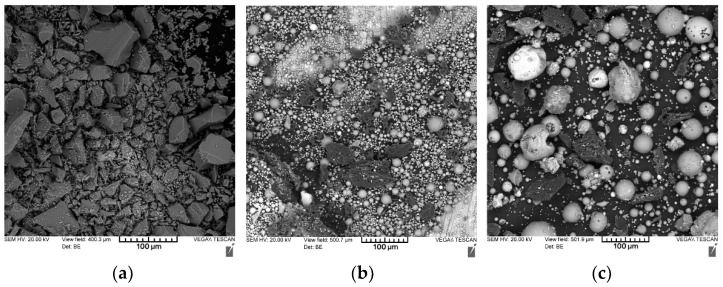
Microstructures of the studied coal energy wastes: (**a**) fuel slag; (**b**) fly ash; (**c**) ASM.

To improve the reactivity of the geopolymer suspension, the aluminosilicate precursors were milled up to passing through a 250 µm sieve. The microstructures of the prepared wastes under study are shown in Figure 4.

The microstructure of the milled fuel slag (a) is represented by particles of an irregular shape with a size of 10–100 µm. The particles are represented by an amorphous material with a smooth vitrified surface. The microstructure of fly ash (b) is mainly represented by hollow aluminosilicate microspheres with a size of 1–50 µm.

These microspheres are formed during high-temperature combustion of coal due to melting and fusing of mineral impurities. The inner part of the ash spheres is mainly filled with nitrogen and carbon dioxide. Microspheres can have both a smooth vitrified surface or a porous spongy structure. The presence of hollow aluminosilicate microspheres in the composition of fly ash explains its lowest density (2051 kg/m^3^) among the studied materials (in comparison, the density of slag is 2356 kg/m^3^ and the density of ASM is 2333 kg/m^3^).

The ash and slag mixture is obtained due to spontaneous mixing of fly ash and fuel slag at the dump. Thus, the microstructure of the milled ASM (c) is represented by a combination of fuel slag particles of an irregular shape and hollow aluminosilicate microspheres. Moreover, in the studied mixture, an increased content of microspheres was observed, and therefore, it can be concluded that there is more fly ash than fuel slag in the studied ASM.

### 3.2. Foaming Mechanisms and Properties of Porous Geopolymers

The following compounds were used as foaming agents: ASD-1-grade spherical dispersed aluminum (powder), 30% hydrogen peroxide solution, and A-grade 10% sodium hypochlorite solution. 

The foaming mechanism when using aluminum powder is explained by its interaction with a sodium hydroxide solution—the main component of an alkaline activator. Aluminum, as an amphoteric metal, interacts with alkali to form hydroxoaluminates and release hydrogen, which has a foaming effect according to Reaction (6):

2Al + 2NaOH + 6H_2_O = 2Na[Al(OH)_4_] + 3H_2_↑ (6)

This reaction does not occur immediately, allowing the control of the foaming process and, accordingly, forming the material without the loss of foaming gas. In addition, the rate of this reaction becomes greater when the raw mixture is heated. 

The foaming mechanism when using concentrated solutions of hydrogen peroxide (usually a 30% aqueous solution) based on its decomposition with the formation of water and oxygen gas foams the raw mixture (Reaction (7)):

2H_2_O_2_ = 2H_2_O + O_2_↑ (7)

The process of foaming when using a 30% aqueous solution of hydrogen peroxide proceeds intensively, starting at the stage of the preparation of the reaction mixture. This factor negatively affects the foaming process, since part of the gas is lost during the raw materials’ mixing. This reaction could be accelerated when the raw mixture is heated. Furthermore, the reaction is accelerated due to the action of transition metal oxides from the coal energy wastes.

The foaming mechanism when using alkaline hypochlorites is based on their properties, especially their thermal instability and decomposition upon heating according to Reaction (8):

2NaOCl = 2NaCl + O_2_↑ (8)

At room temperature, this reaction is very slow, but it decomposes very easily when heated above 70 °C. This decomposition leads to the release of oxygen, which has a foaming effect.

**Figure 5:**
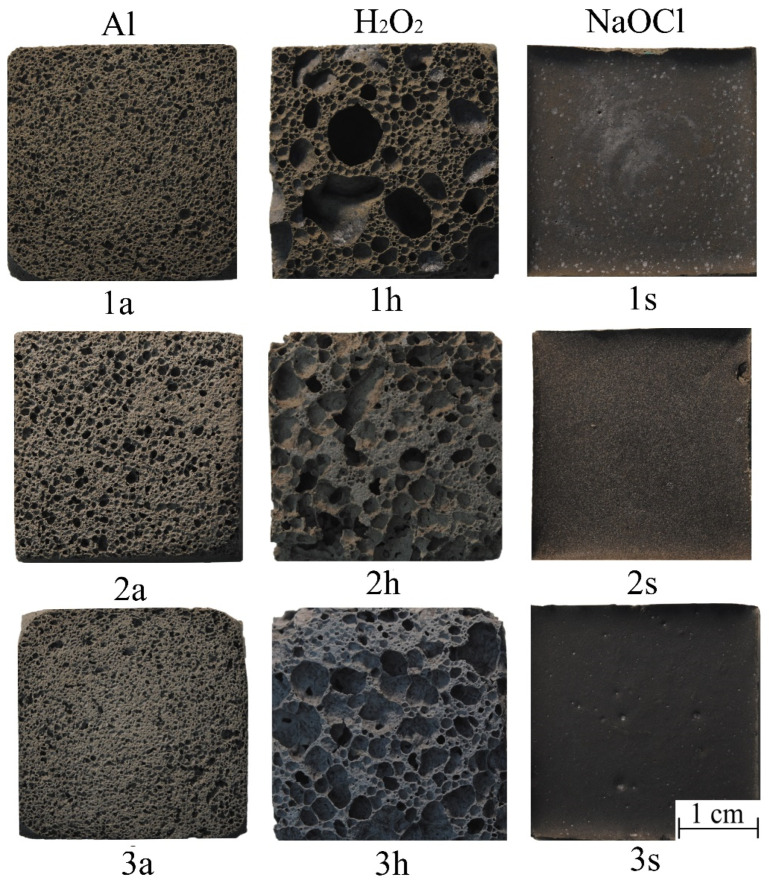
Structure of synthesized porous geopolymers: 1—fly ash, 2—slag, 3—ash and slag waste; a—aluminum powder, h—hydrogen peroxide, s—sodium hypochlorite.

Based on the presented component mixtures (Table 1) and the developed technology (Figure 1), foamed geopolymer samples were synthesized. Their internal structure is shown in Figure 5.

**Figure 6:**
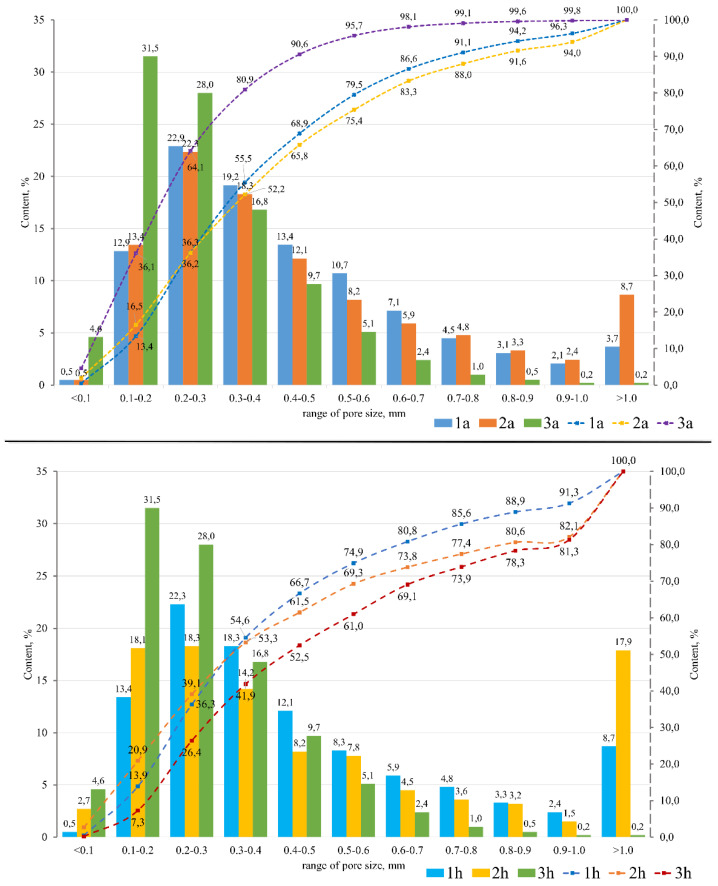
Histograms and integral curves of pore size distribution ranges: 1a—fly ash + aluminum powder; 1h—fly ash + hydrogen peroxide; 2a—fuel slag + aluminum powder; 2h—fuel slag + hydrogen peroxide; 3a—ASM + aluminum powder; 3h—ASM + hydrogen peroxide.

According to Equations (2)–(5), the main characteristics of the synthesized porous geopolymers were calculated (Table 4). Figure 6 shows the histograms of the pore size distribution in the studied samples. Since porous geopolymer materials with sodium hypochlorite as the foaming agent are a monolithic structure, it was decided not to analyze its pore size distribution ranges.

The porous geopolymers of the 1a, 2a, and 3a compositions (with aluminum powder as a foaming agent) have a uniformly distributed porous structure, which has a positive effect on the material properties. In all synthesized samples, macropores in the size range of 0.1–0.5 mm predominate. Composition 3a has the smoothest vertical growth of the integral curve with a significant predominance of a pore size up to 0.5 mm (90.5%). Thus, this composition has the most uniform porous structure among all the others. The integral curves of the 1a and 2a compositions have a smoother horizontal growth, which indicates an uneven distribution of the pore sizes in these compositions.

The porous geopolymers of the 1h, 2h, and 3h compositions (with the 30% aqueous hydrogen peroxide solution as the foaming agent) have an unevenly distributed porous structure with a predominance of the pore size range of 0.1–0.5 mm (60–75%) and a significant amount of macropores larger than 1 mm (8.7–18.7%). Samples with the 30% hydrogen peroxide aqueous solution have the lowest compressive strength among all the foaming agents used, which is confirmed by the data in Table 4. The compressive strength of these samples is almost two-times lower than Samples 1a, 2a, and 3a with the addition of the aluminum foaming agent. Such a strength reduction is directly connected to the amount of large pores, which also have a great negative effect on the stability of the properties.

Porous geopolymers of the 1s, 2s, and 3s compositions (with the 10% sodium hypochlorite solution as the foaming agent) have a monolithic structure and do not have pores. In this regard, they have the highest strength, density, and thermal conductivity among all the studied compositions. The absence of pores is most likely due to the low concentration of hypochlorite ions in the geopolymer suspension and, correspondingly, very low foaming activity.

**Figure 7:**
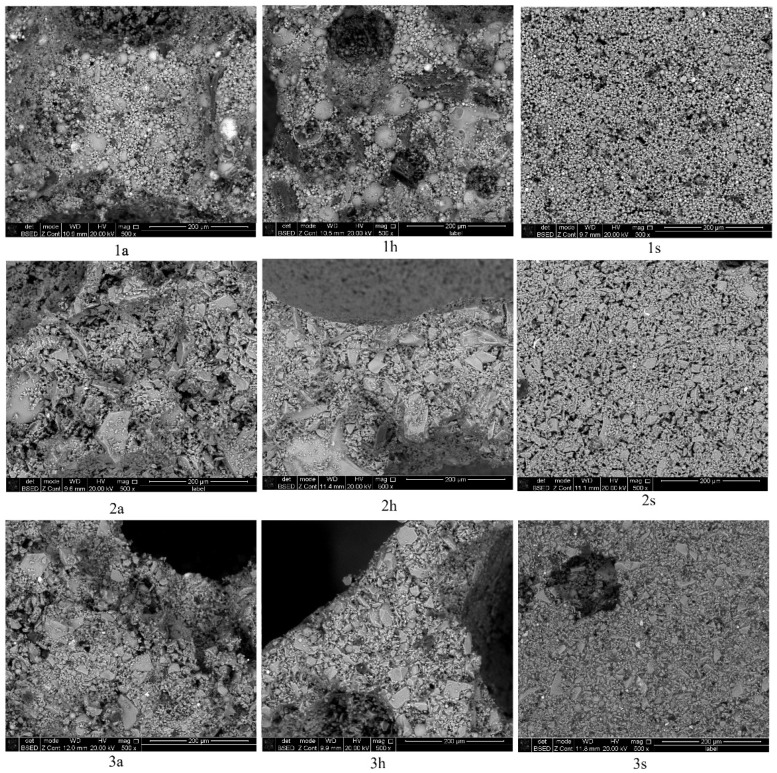
Microstructure of the studied geopolymers: 1—fly ash, 2—slag, 3—ash and slag mixture; a—aluminum powder, h—hydrogen peroxide, s—sodium hypochlorite.

The microstructures of the porous geopolymers based on the coal combustion wastes with the use of foaming additives are shown in Figure 7.

The analysis of the structure of the interpore walls of the geopolymers based on fly ash showed the presence of hollow aluminosilicate microspheres with sizes from 2 to 50 µm. The microstructure of the samples based on slag is represented by particles of an irregular shape with sizes varying from 2 to 150 µm. In samples based on ash and slag mixture, there are inclusions of glass spherical formations with particle sizes from 5 to 20 µm. In samples of Series 1s, microcracks ranging in size from 2 to 10 μm were observed. Their presence negatively affects the ultimate strength of the samples, which is confirmed by the values obtained in Table 3. Thus, the geopolymer is a polycrystalline material consisting of aluminosilicate particles of irregular and spherical shapes, bonded together by the reaction products of alkaline and aluminosilicate components.

Thus, the optimal foaming agent is aluminum powder, which allowed obtaining the most uniform cellular structure of the foamed material, which has a positive effect on its properties. Hydrogen peroxide, despite its high foaming activity, did not allow obtaining a uniform distribution of the macropores. The sodium hypochlorite solution did not show foaming activity, which is associated with its low concentration. Therefore, in the future, it would be promising to study the foaming activity of more concentrated solutions of sodium hypochlorite.

The type of coal energy waste has a lesser effect on the structure and properties of the synthesized geopolymers. Samples based on ash and slag mixture have the highest density. This is probably due to contamination of ASM at the dump, as described above. Samples based on fly ash have a lower density and a fairly uniform structure. However, fly ash, unlike fuel slag and ASM, is widely used in other industries, especially in the production of Portland cement. Samples based on fuel slag have the best thermal insulation characteristics. This is due to the fact that slag has the largest proportion of the amorphous phase among the studied coal energy wastes, and therefore, it is the most reactive. However, from an economic and environmental point of view, it is most expedient to use ASM as the large-tonnage waste that has the greatest potential environmental hazard. Therefore, in the future, it is advisable to study ASM’s purification and enrichment methods, as well as methods for the intensification of foaming and geopolymer structure formation.

Thus, the optimal coal energy waste for the synthesis of porous geopolymers is fuel slag, and the optimal foaming agent is aluminum powder. The use of such a ratio of components makes it possible to obtain a porous geopolymer material with a predominant pore size of 0.1–0.5 mm, a density of 590 kg/m^3^, a compressive strength of 2.37 MPa, and a thermal conductivity of 0.1385 W/(m·K). The developed geopolymer materials can be used in the construction of roads, buildings, structures, in the design and construction of industrial equipment for various purposes, etc. The promising application of such materials is the construction of a frost-protective layer for roadways in extreme climatic conditions because of the high frost resistance of the synthesized materials.

## 4. Conclusions

In this paper, the possibility of using coal energy waste (fly ash, boiler (fuel) slag, ash and slag mixture (ASM)) in the synthesis of porous geopolymer materials was considered. According to the chemical composition, all the wastes were represented by SiO_2_ (46.85–56.12 wt. %), Al_2_O_3_ (18.78–21.97 wt. %), Fe_2_O_3_ (8.67–10.74 wt. %), and small amounts of Na_2_O, K_2_O, CaO, MgO, TiO_2_, and others. The X-ray phase analysis showed a high presence of an amorphous phase in all the studied wastes. The largest amount of the amorphous phase (more than 98%) was contained in the fuel slag. The largest amount of the crystalline phase (more than 27%) was contained in the ASM, which was associated with its contamination during storage on the dump. The microstructure and granulometric composition of the coal energy wastes revealed that fly ash and ASM contained hollow aluminosilicate microspheres formed during high-temperature coal combustion. Fly ash is a highly dispersed material, consisting almost entirely of these microspheres with a size of less than 0.063 mm. ASM is a polydisperse material with a predominance of particles with a size more than 1.0 mm. The fuel slag was not studied, since it is represented by big, vitrified pieces larger than 5 cm.

Raw mixtures based on various wastes from coal energy production (ash, slag, ash and slag mixture) and foaming agents (aluminum powder, 30% hydrogen peroxide solution, 10% sodium hypochlorite solution) were chosen, and foamed geopolymer materials were synthesized. It was discovered that the optimal foaming agent was aluminum powder. It allowed obtaining a uniform cellular structure, which favorably affected the performance properties of the foamed geopolymer material. The solution of hydrogen peroxide, despite the high foaming activity, did not allow obtaining a uniform cellular structure; the pore size in the samples varied in a wide range, which negatively affected the thermal insulation and mechanical properties of the geopolymer material. The sodium hypochlorite solution did not show foaming activity, which was due to the low concentration of the solution used. 

The type of coal energy waste had a lesser effect on the structure and properties of the synthesized geopolymers. Properties decreased in the line “fuel slag—fly ash—ASM”. The samples based on fuel slag had the best characteristics due to the fact that slag has the largest proportion of the amorphous phase and, therefore, it is the most reactive. However, as long as ASM is the large-tonnage waste that has the greatest potential environmental hazard from an economic and environmental point of view, it is promising to study the ASM purification and enrichment methods, as well as methods for the intensification of foaming and a geopolymer structure formation. 

The optimal composition of the geopolymer materials was obtained on the basis of fuel slag and aluminum powder as the foaming agent. Such a combination allowed obtaining the uniform cellular structure of the material with a predominant pore size of 0.1-0.5 mm, a density of 590 kg/m^3^, a compressive strength of 2.37 MPa, and a thermal conductivity of 0.1385 W/(m·K). The developed geopolymer materials can be used in the construction of roads, buildings, structures, and in the design and construction of industrial equipment, especially in harsh conditions.

## Figures and Tables

**Table 1:** Component composition of the raw mixture, wt. %.

	Precursor (Type of ASW)	NaOH (Powder)	Water	Waterglass	Aluminum Powder, over 100	Hydrogen Peroxide, over 100	Sodium Hypochlorite, over 100
1a	70.0 (fly ash)	2.5	5.0	22.5	2.0	–	–
1h	70.0 (fly ash)	2.5	5.0	22.5	–	2.0	–
1s	70.0 (fly ash)	2.5	5.0	22.5	–	–	2.0
2a	70.0 (slag)	2.5	5.0	22.5	2.0	–	–
2h	70.0 (slag)	2.5	5.0	22.5	–	2.0	–
2s	70.0 (slag)	2.5	5.0	22.5	–	–	2.0
3a	70.0 (ASM)	2.5	5.0	22.5	2.0	–	–
3h	70.0 (ASM)	2.5	5.0	22.5	–	2.0	–
3s	70.0 (ASM)	2.5	5.0	22.5	–	–	2.0

**Table 2:** Chemical composition of coal energy wastes.

	SiO_2_	Al_2_O_3_	Fe_2_O_3_	MgO	Na_2_O	K_2_O	CaO	TiO_2_	MnO	P_2_O_5_	SO_3_	LOI
Fly ash	46.85	20.58	8.67	1.30	0.88	3.33	2.03	0.78	0.06	0.14	0.43	14.95
Slag	56.12	21.97	10.74	1.97	1.09	3.57	2.93	0.92	0.13	0.12	0.02	0.42
ASM	51.23	18.78	10.27	2.08	0.92	3.04	3.10	0.78	0.13	0.13	0.31	9.23

**Table 3:** Quality indicators of the studied raw materials.

Quality Indicator	Optimal for Binder Materials	Slag	Fly Ash	ASM
Silicate modulus	1.5–3.6	1.716	1.602	1.763
Basicity modulus	> 1.0	0.122	0.112	0.130
Quality coefficient	1.5–2.5	0.471	0.502	0.461

**Table 4:** Medium characteristics of the synthesized samples.

	Density, kg/m^3^	Compressive Strength, MPa	Porosity, %	Thermal Conductivity, W/(m·K)	Mass Reduction after Curing, %
1a	590 ± 4	2.03 ± 0.07	71.24 ± 0.21	0.1420 ± 0.0038	24.31 ± 0.21
2a	590 ± 23	2.37 ± 0.02	74.82 ± 0.99	0.1385 ± 0.0039	20.48 ± 0.74
3a	548 ± 14	1.32 ± 0.02	76.48 ± 1.75	0.1286 ± 0.0031	30.80 ± 0.44
1h	354 ± 18	1.19 ± 0.06	82.75 ± 1.86	0.0856 ± 0.0009	22.19 ± 0.63
2h	373 ± 7	1.24 ± 0.03	83.99 ± 2.00	0.0878 ± 0.0018	21.94 ± 0.36
3h	335 ± 24	1.06 ± 0.04	85.61 ± 0.13	0.0793 ± 0.0008	25.01 ± 0.48
1s	1438 ± 34	4.00 ± 0.06	29.91 ± 0.56	0.3552 ± 0.0027	24.68 ± 0.99
2s	1504 ± 41	6.46 ± 0.08	35.85 ± 0.74	0.3629 ± 0.0058	20.64 ± 0.87
3s	1552 ± 16	4.96 ± 0.09	33.40 ± 0.25	0.3751 ± 0.0039	25.78 ± 0.34

## Data Availability

All data presented in this study are included in the published article.
